# Conservative Management of Gunshot Wound to Anterior Abdomen in a Resource-Poor Setting in the Caribbean

**DOI:** 10.7759/cureus.16789

**Published:** 2021-07-31

**Authors:** Shariful Islam, Malini Ramnarine, Anthony Maughn, Kiran Chandolu, Vijay Naraynsingh

**Affiliations:** 1 General Surgery, San Fernando General Hospital, San Fernando, TTO; 2 Clinical Surgical Sciences, The University of the West Indies, St. Augustine, TTO; 3 Radiology, San Fernando General Hospital, San Fernando, TTO; 4 Surgery, Medical Associates Hospital, St. Joseph, TTO

**Keywords:** gunshot wound to the abdomen, anterior penetration, selective non-operative management, penetrating trauma, wounds and injuries

## Abstract

Previously, the management of gunshot wounds (GSWs) to the anterior abdomen required exploratory laparotomy; however, this was associated with a considerable number of non-therapeutic surgeries. The use of non-operative management (NOM) of GSW to the abdomen is controversial, with many surgeons sceptical to accept this into their practice. The NOM of GSW to the abdomen employed in a selected group of patients has been shown to be safe and acceptable. Penetrating GSW to the thoraco-abdomen, back and lateral abdomen has been the most successful compared to the anterior penetrating wound. Most of the anterior GSWs to the abdomen are associated with viscus injury and require exploratory laparotomy. We report the case of a 58-year-old male who presented with a single GSW to the epigastrium with a contrast computed tomography scan demonstrating grade 3 liver lacerations, contusion to the right adrenal gland, with moderate free fluids in the retroperitoneum and the pelvis. The patient was haemodynamically stable and managed successfully with NOM. It is one of the safe routes of anterior penetration of GSW to the abdomen and treated with conservative management.

## Introduction

Penetrating injury to the abdomen is the most common surgical admission. Until today, the standard management of gunshot wounds (GSWs) to the abdominal cavity is mandatory laparotomy [1]. The idea of selective non-operative management (SNOM) of penetrating injuries to the abdomen was first coined by Shaftan et al. in 1969 [2]. However, during 1990, serious concern was raised for mandatory laparotomy for all penetrating abdominal wounds. Thereafter, it slowly gained acceptance amongst trauma surgeons all over the world. SNOM is now often conducted in most trauma centres across the world in haemodynamically stable patients without any signs of peritonitis or evisceration. However, this is only true for abdominal stab wounds [2-4] and not for the GSW into the abdomen. Most surgeons still prefer exploratory laparotomy for the GSW into the abdomen. The reasons for this preference are that GSW is often associated with a high incidence of intra-abdominal injuries, the morbidity and mortality of missed injuries [4,5]. Despite documented safety of SNOM of abdominal GSW from both retrospective and prospective studies, it remains controversial among surgeons. We document a case of GSW with anterior penetration of the abdomen and managed it successfully with non-operative management (NOM).

## Case presentation

**Figure 1:**
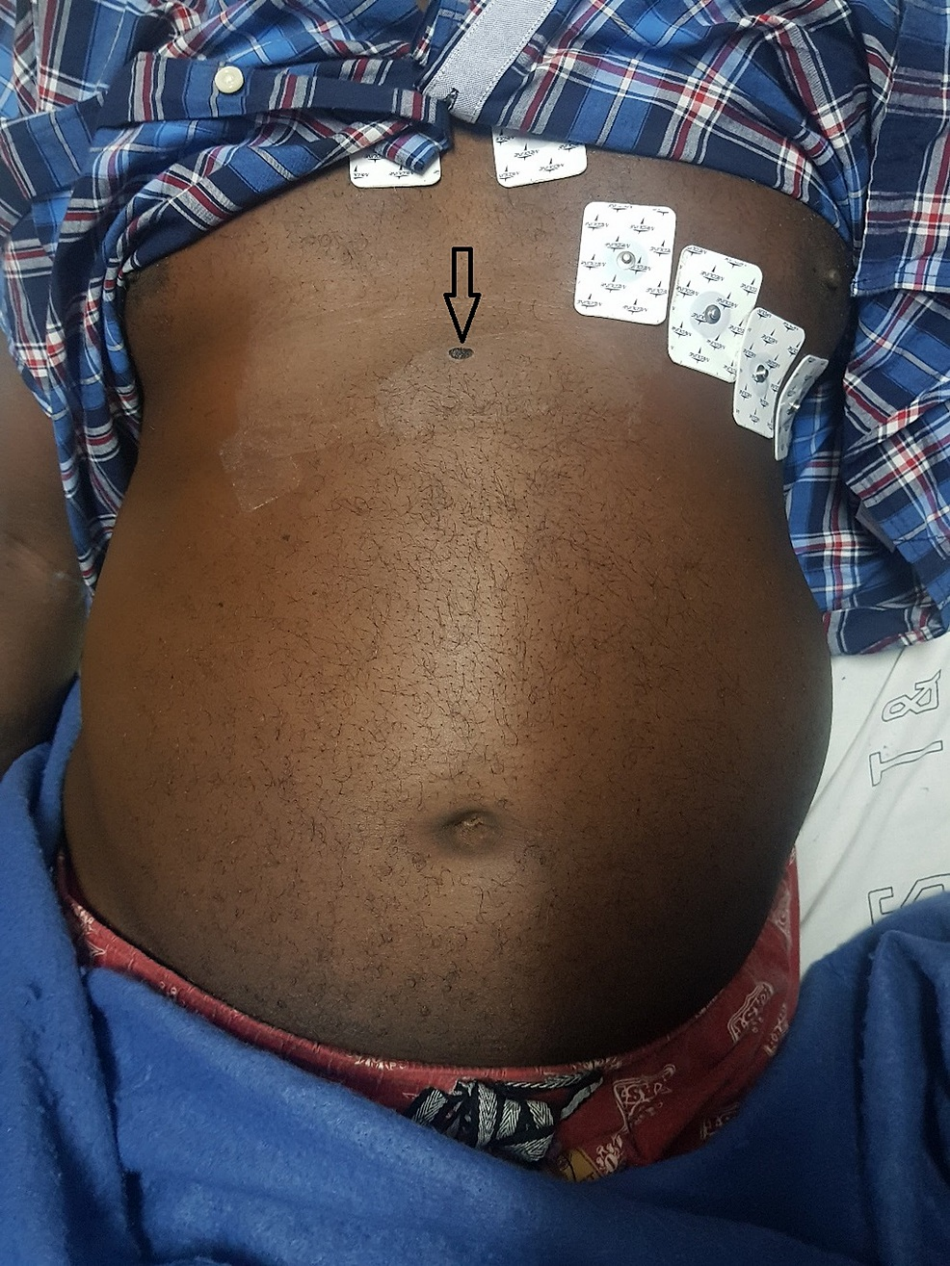
Entry point of a gunshot to the epigastrium (black arrow)

A 58-year-old male, known hypertensive presented with a single gunshot to the epigastrium for six hours (Figure 1). He experienced epigastric and right upper abdominal pain with no haemoptysis or per rectal bleeding. On examination, the patient was conscious and had no cardiopulmonary distress. His mucous membranes were moist and pink. His vital signs were all within normal range. Abdominal examination revealed a single entry wound into the epigastrium but no exit wound was noted. There was mild tenderness in the epigastric and right upper quadrant with no distension, guarding or rebound tenderness. Digital rectal examination revealed no blood on gloves and there was no microscopic or macroscopic haematuria noted. His haemoglobin levels were stable and they varied between 14.1mg/dL and 13.2 mg/dL. Erect chest x-ray was negative for pneumo-peritoneum, pneumothorax or haemothorax and abdominal x-rays showed no significant pathologies. FAST ultrasound scan was negative for free intra-peritoneal or pelvic fluids.

**Figure 2:**
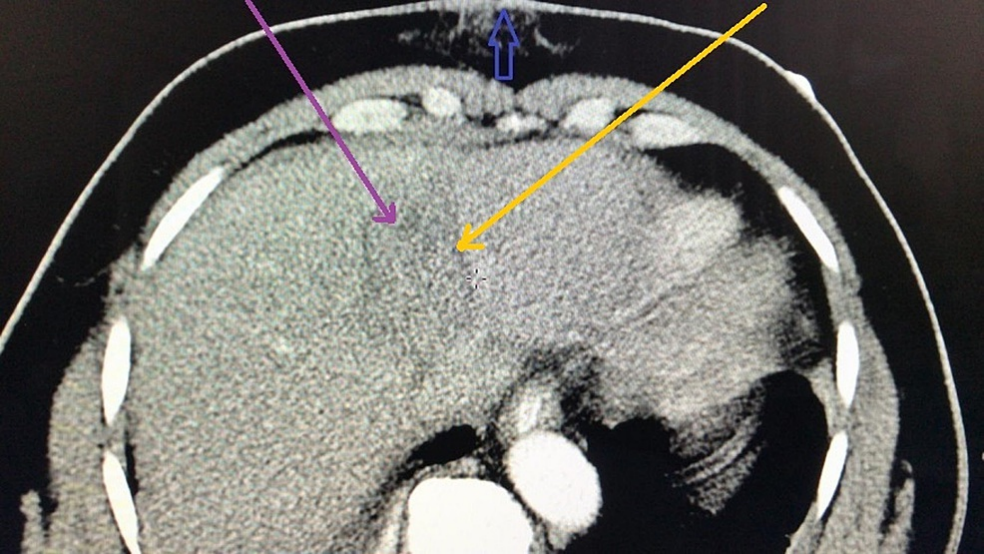
CT scan of abdomen and pelvis with intravenous contrast showing a grade Ⅲ liver injury through segment Ⅷ (blue arrow showing penetration of the skin and subcutaneous tissue of anterior abdominal wall; the purple and yellow arrows showing the non-enhancing low attenuation area with associated intra-parenchymal haematoma)

**Figure 3:**
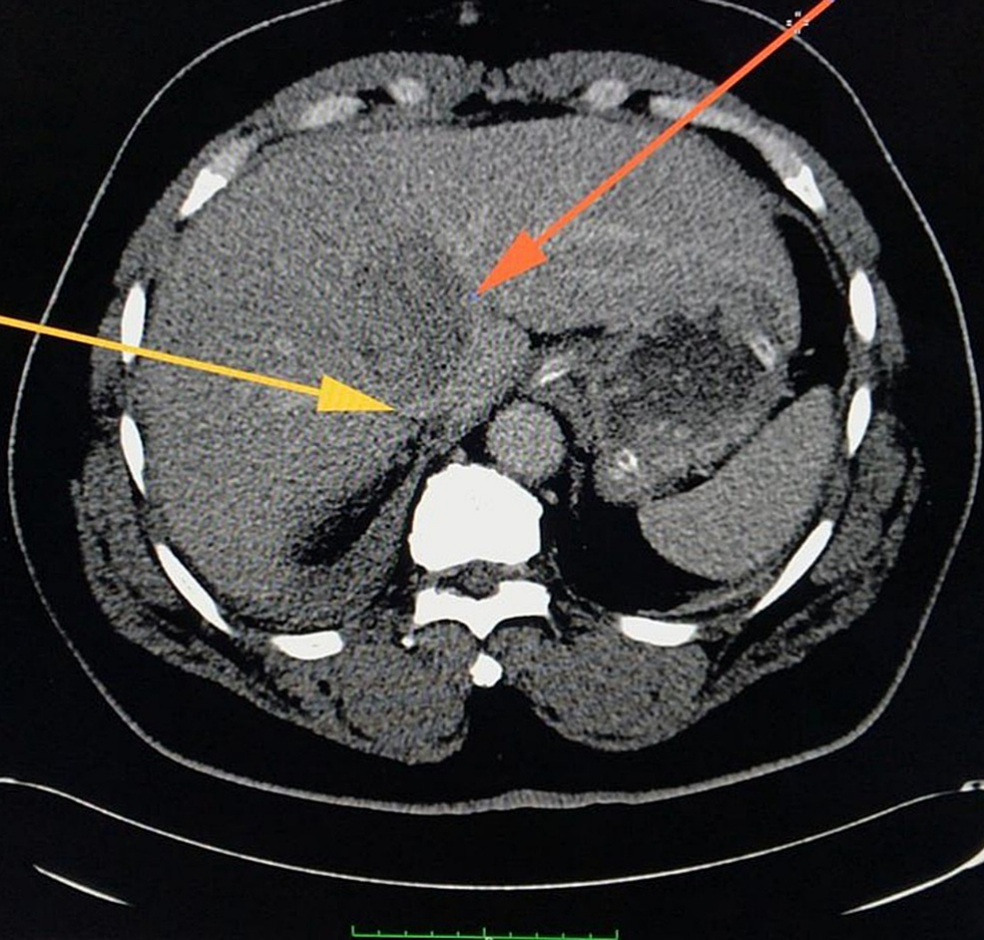
CT scan of abdomen and pelvis with intravenous contrast showing haematoma (orange arrow) compressing the inferior vena cava (yellow arrow) with no extravasation of blood

**Figure 4:**
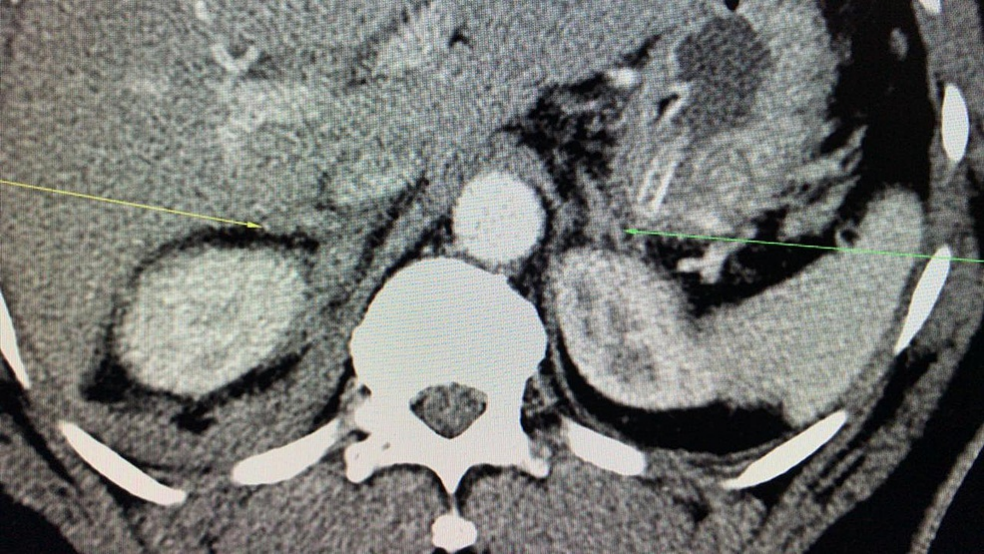
CT scan of abdomen and pelvis with intravenous contrast showing contused right adrenal gland ( yellow arrow) and normal left adrenal with fatty hilum (green arrow)

**Figure 5:**
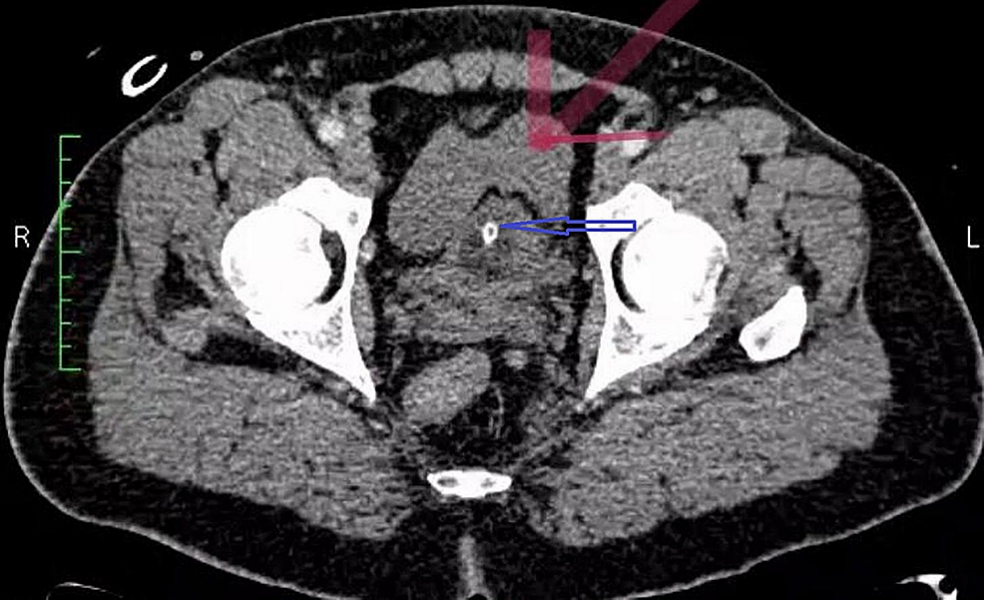
CT scan of abdomen and pelvis with intravenous contrast showing free fluid in the pelvis (red arrow) with urinary catheter in situ (blue arrow)

**Figure 6:**
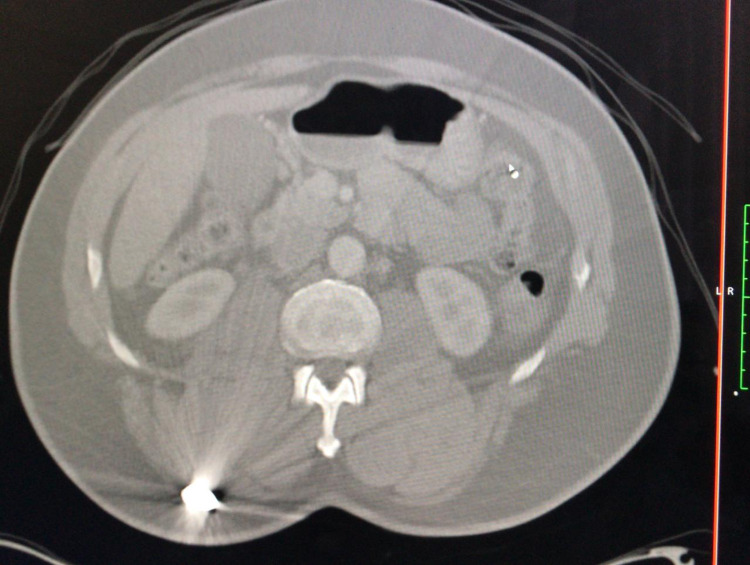
The bullet lodge in the soft tissue of the back at the level of the right first lumbar vertebra

A contrast-enhanced computed tomography (CT) scan of the abdomen and pelvis showed a grade Ⅲ liver injury through segment Ⅷ measuring 7.1 cm x 3.9 cm (Figure 2). There was no free intra or retroperitoneal air noted on the scan. The inferior vena cava was compressed by the haematoma but there was no blush or extravasation seen (Figure 3). The right adrenal gland was also contused with loss of fatty hilum (Figure 4). A small amount of free fluid was also noted in the pelvis (Figure 5). The bullet was seen within the soft tissue of the right flank at the level of the first lumbar vertebrae (Figure 6).

This patient remained stable throughout the admission. The patient was managed successfully by conservative treatment with serial abdominal examinations and haemoglobin levels every four hours in the high dependency unit. The abdominal examinations were performed by the same surgeon in the first 24 hours and thereafter by the on-call senior resident. The patient was discharged home on day 3 post-admission. At one year of follow-up, the patient was doing well with no further complaints.

## Discussion

The NOM of blunt abdominal trauma and stab wounds to the abdomen is well recognized and accepted in haemodynamically stable patients [6]. The current standard of care for GSW to the abdomen is controversial, with exploratory laparotomy considered the standard previously. Exploratory laparotomy for GSW to the abdomen was associated with a negative laparotomy rate of 20% and a complication rate of 20% in this group [7]. Non-therapeutic and negative laparotomies are linked with an increased hospital stay (5-9 days), cost [6] and morbidity (22%-41%) due to postop ileus, pneumonia, surgical site infections and thromboembolic events [8].

Shaftan in 1960 was one of the first to introduce the concept of NOM for penetrating abdominal injuries when he reported a 34.1% negative laparotomy rate [7], which was later endorsed by Nance and Cohn in 1969 for stabbing abdominal injuries [9]. Many prospective and retrospective studies have evaluated the use of selective NOM of GSW to the abdomen concluding favourable outcomes.

The subset of patients selected for NOM is determined by the patient’s conscious level, clinical examination, and CT findings. In patients with depressed conscious level, a severe injury requiring endotracheal intubation and sedation, in whom clinical examination will be unreliable, NOM is contraindicated. The sensitivity, specificity and accuracy of clinical examination in determining the need for urgent surgical intervention has been questioned [10]. Velmahos reported a sensitivity of 100% and specificity of 95.3% [10] for clinical examination and Demetriades found the sensitivity of 97.1% [11] supporting it as an accepted method for continued evaluation during NOM of GSW to the abdomen.

Abdomino-pelvic CT is recommended as a prerequisite for NOM of GSW to the abdomen as it gives critical information in making the decision for the treatment pathway. Advantages of CT scan include determining whether the peritoneum is breached, a high sensitivity (90%) for detected intra-peritoneal injuries (solid organ and intestinal) and also characterising the trajectory of the bullet [8]; however, it is not accurate for detecting diaphragmatic [9].

Demetriades et al. in their series of 41 patients with GSW to the abdomen started NOM, with seven patients requiring delayed surgery [11]. Demetriades concluded that selective NOM of GSW to the abdomen is safe and effective as he reported no serious morbidity and no mortality in his prospective series [11]. In 2001, Velmahos et al. published a retrospective review of 792 patients with GSW to the abdomen that was subjected to NOM. He reported that 10% of the patients in this series required delayed laparotomy and 90% were treated successfully with NOM, which was associated with a significant decrease in hospital stay and cost [12]. The complication rates documented with NOM of GSW to the abdomen was 13% in a systematic review by Rawahi et al. [8], which is almost 50% less than that reported for mandatory laparotomies.

There is also a debate on how long patients should be observed if they undergo NOM in penetrating abdominal injuries. Many studies agree that in the presence of minimal tenderness on abdominal examination for 24 hours, the patient can be safely discharged. This recommendation was supported by Velmahos et al., as he found observation beyond 24 hours is not necessary for stable, asymptomatic patients that tolerate diet [12]. We could possibly discharge our patient after 24 hours of observation. However, we were a bit cautious as it was our first case of anterior penetration of GSW managed conservatively. For the first 24 hours we kept our patient nil by mouth followed by cleared fluids graduated to a soft diet and discharged home on day 3.

According to the Eastern Association for the Surgery of Trauma (EAST) guideline for penetrating abdominal trauma patients, NOM should be contraindicated in patients with diffuse peritonitis, haemodynamic instability, unreliable or unable to perform adequate physical examination due to any circumstances. Criteria for safe SNOM for the GSW to the abdomen are patients who arrive and remain in a stable haemodynamic condition, no signs of diffuse peritonitis, the ability to perform a timely reliable clinical and laboratory examination by a trained surgical team, with timely available theatre spaces. Any changes in the clinical or laboratory parameters of the patient will indicate the need for immediate laparotomy. This approach can minimize not only unnecessary laparotomy but also reduce the risk of unnoticed injuries [13,14].

A correct approach for SNOM for GSW to abdomen would require monitoring vitals, serial haematocrit and clinical examinations (abdominal pain, fever and change in level of consciousness) every four hours by a multidisciplinary team (managing surgeon, nurses, and laboratory technicians, anaesthetist, interventional radiologist). Many studies have highlighted the importance of subsequent clinical examination preferably by the same surgical team who made the initial choice of NOM. It was also equally important to carry out this treatment in specialized centres, which have all the above facilities and the capability to deal with these patients without causing any harm to the patients [14-17]. Our patient was managed in the HDU for the first 24 hours and thereafter in the general surgical ward. Serial clinical examinations and haematocrit were performed every four hours by the same surgeon for the first 24 hours and thereafter by the on-call senior surgical resident.

The knowledge of the subdivisions of the abdomen is of great importance, as the success of SNOM of penetrating GSW depends on regions of entry into the abdomen. The abdomen is subdivided into four regions: a) the anterior abdomen-bounded by the costal margins superiorly, the anterior axillary lines laterally, and the inguinal creases inferiorly b) the thoraco-abdomen-bounded inferiorly by the costal margin and superiorly by the nipples (or tips of the scapulae), c) bilateral flanks bounded superiorly by the costal margins, inferiorly by the iliac crests, anteriorly by the anterior axillary lines and posteriorly by the posterior axillary line and d) the back bounded by the inferior scapular tips superiorly, the iliac crests inferiorly and the posterior axillary lines laterally [18].

Rawi et al. in their systematic review noted that the risks of failure of SNOM were lowest in studies that evaluated patients with right thoraco-abdomen (3.4%; 95% CI = 0%-7.0%; I2 = 0%; homogeneity p = 0.45), flank (7.0%; 95% CI = 3.9%-10.1%) and back (3.1%; 95% CI = 0%-6.5%) GSWs and highest in those that evaluated patients with anterior abdomen (13.2%; 95% CI = 6.3%-20.1%) GSWs. In patients who underwent mandatory abdomino-pelvic computed tomography (CT), the pooled risk of failure was 4.1% versus 8.3% in those who underwent selective CT (p = 0.08). The overall sample-size-weighted mean hospital length of stay among patients who underwent SNOM was six days versus 10 days if they failed SNOM or developed an in-hospital complication [8]. Similar results were also noted in a more recent systematic review by Silva [10]. Thus, a selected group of patients with GSW to the abdomen can be successfully managed by SNOM. However, this can only be safely applied in a selected group of patients in centres with the capability of necessary laboratory tests, imaging facility, availability of blood and blood products, timely available theatre spaces and in the presence of trained multidisciplinary staff.

## Conclusions

In stable patients without haemorrhage or evisceration the selective use of NOM in stab wounds to the abdomen is more accepted and less debated; however, this notion is not embraced in GSW to the abdomen as the outcomes of missed injuries can be unfavourable. Institutions should develop protocols and train required staff (surgeons, nurses and anaesthetist) in NOM of GSW to the abdomen to reduce unwarranted laparotomies in a selected group of patients without increasing morbidity and mortality.
